# Scoring the physical frailty phenotype of patients with heart failure

**DOI:** 10.1002/jcsm.12883

**Published:** 2021-11-21

**Authors:** Masaaki Konishi

**Affiliations:** ^1^ Department of Medical Science and Cardiorenal Medicine Yokohama City University Graduate School of Medicine Yokohama Japan

**Keywords:** Ageing, Frailty, Heart failure

Diagnosing physical frailty in patients with heart failure (HF) is often difficult. Even though physicians are increasingly having these so‐called ‘physically frail’ patients with HF, it may occasionally be challenging to apply some commonly used concepts of frailty, such as the frailty phenotype proposed by Fried *et al*.^1^ in patients with HF. First, exhaustion, one of the five major phenotypes of physical frailty (shrinkage/weight loss, weakness, exhaustion, slowness, and low activity), is observed in most patients with HF.^2^ This indicates that the presence or absence of exhaustion is not useful in differentiating frail from non‐frail patients when HF is the main diagnosis of the patient. Second, it is often difficult for clinicians to determine the presence or absence of shrinkage or weight loss, another important phenotype of physical frailty, in patients with HF. This is because HF is a clinical syndrome that maintains both congested and decongested states.

A multicentre prospective cohort study to develop frailty‐based prognostic criteria in heart failure patients (FLAGSHIP) by Yamada *et al*. took a great step forward in this difficult situation.^3^ FLAGSHIP was designed to enrol ambulatory patients admitted for acute HF.^4^ The authors should be commended for conducting this study and establishing a new scoring system for physical frailty in HF. The scoring system of physical frailty proposed by Yamada *et al*. is an original and creative development based on the cut‐off value according to HF‐specific outcomes: the composite of HF rehospitalization and all‐cause death within 2 years. The authors collected data including those on items related to the frailty phenotype; known prognostic factors of HF; and potential components of frailty, such as cognitive function, depression, and anorexia. We commend the authors for enrolling enough patients (2884 patients with HF) within a study period of 40 months from 30 collaborating hospitals nationwide in Japan. This study was characterized by two methods for analysing the results. First, establishing the cut‐off value of each component of physical frailty (weight loss, weakness, exhaustion, slowness, and low activity) is an original approach. The cut‐off value was determined using Youden's index with the receiver‐operating characteristic curve to predict the outcome. As a result, the cut‐off value for weakness was grip strength 30.0 kg for men and 17.5 kg for women and that for slowness was walking speed 0.98 m/s for both sexes. Interestingly, the cut‐off value of weakness was quite similar to that in the original report by Fried *et al*.^1^: 29–32 kg for men and 17–21 kg for women. However, the cut‐off value of slowness was higher than that in the original report: 0.65–0.76 m/s (6–7 s to walk 15 feet) for both sexes. This difference is understandable because the cut‐off value established by Fried *et al*. was derived from the lowest 20% value for older adults but is not outcome oriented. Second, Yamada *et al*. assigned a score for each component of physical frailty: 5 points for weakness, 4 for slowness, 3 for physical inactivity, 2 for exhaustion, and 0 for weight loss, whereas Fried *et al*. assigned a score of 1 point for each component. These values facilitated simple quantification of physical frailty and precise prognostication for patients with HF.

The results of the study by Yamada *et al*. may offer promising leads for future research. First, the target population was broad. With population ageing worldwide, frail patients now account for most patients with HF in daily practice.^5^ Second, there is immense room for improvement for the targeted patients. Frailty is associated with limitations in drug selection and invasive therapies and subsequent poor outcomes, including hospitalization for HF and death.^6^ However, researchers have excluded elderly patients and patients with comorbidities from clinical trials on HF; thus, data on such populations are scarce. Furthermore, there are few effective drug treatments for HF with preserved ejection fraction, which are common in the elderly. Identifying which components of frailty correlate with poor prognosis would help us focus on the target population to be intervened. Finally, scoring the severity of frailty in HF may allow us to repeat the assessment and effective monitoring of patients.

The following points should be noted when interpreting the results of their study: First, they assessed frailty using criteria different from those commonly used. It is not possible to compare the prevalence of frailty in this study with other diseases, with healthy elderly people, or with other cohorts of HF. This may also be aside from the original intention of diagnosing frailty, which was to prevent disability.^7^ The patients included were hospitalized patients with acute HF, in contrast to those for whom frailty is usually assessed in a chronic, stable state. The lower prognostic importance of weight loss observed in this study may also be owing to the unreliability of information on weight gain or loss at the time of hospitalization. However, hospitalization for HF may be the first and most important event that involves many elderly patients in medical care. Inpatient settings also have the advantage of allowing a more comprehensive evaluation than outpatient settings. When it is difficult to assess body weight, measurement of skeletal muscle mass may be an option. Sarcopenia (loss of muscle mass plus muscle strength and/or physical performance) is considered the core pathophysiology of frailty and was associated with poor prognosis of patients with HF.^8^, ^9^ Yamada *et al*. used the Performance Measure for Activities of Daily Living‐8 (PMADL‐8) score as a measure of exhaustion. Despite its name, it was originally developed to assess exercise tolerance in HF along with the New York Heart Association classification. In Fried's original text, exhaustion should be self‐reported; self‐reported exhaustion is associated with the stage of exercise reached in a graded exercise test as an indicator of VO_2_ max, and thus, the use of PMADL‐8 as an objective measure of exhaustion is justified. The index used for low activity was the Self‐Efficacy for Walking‐7 questionnaire, and the validity of its application for patients with HF is currently unknown. In fact, although several meta‐analyses for frailty in HF have been published,^10^, ^11^, ^12^ they focus on the comparison between Fried and non‐Fried criteria and do not discuss the details of each component of Fried's frailty phenotypes. Yamada *et al*. focused on physical frailty in this study. However, multidisciplinary assessment has recently become important when considering frailty in patients with HF. Social and cognitive frailty are closely associated with physical frailty.^13^, ^14^, ^15^, ^16^

The results of the study by Yamada *et al*.^3^ will be useful for future interventional studies. Nutrition and exercise therapy are major candidates for interventional studies of physical frailty in patients with HF. A randomized controlled trial of 645 hospitalized patients with HF at risk of malnutrition has recently been published.^17^ Inpatients were randomized to individualized nutritional support or standard hospital food; the individualized nutritional support group received targeted energy (using the Harris–Benedict equation or indirect calorimetry) and protein (1.2–1.5 g/kg/day), with oral nutritional support when intake was inadequate. The primary endpoint of death at 30 days was observed in 8.4% of the individualized nutritional support group and 14.8% of the standard hospital diet group members, with an odds ratio of 0.44 for death, indicating the usefulness of individualized nutritional support. The Rehabilitation Therapy in Older Acute Heart Failure Patients (REHAB‐HF) trial was a multicentre, randomized, controlled trial involving 349 mostly frail or prefrail patients with HF.^18^ An early and tailored cardiac rehabilitation approach resulted in greater improvement in physical function than usual care for hospitalized patients with HF.

In conclusion, the study by Yamada *et al*.^3^ provides an insight into the assessment of frailty in HF and a step towards better therapeutic strategies. Effective intervention studies will hopefully be designed based on the results of this study.

## Conflict of interest

Masaaki Konishi reports no conflict of interest.

## Funding

No funding involved.
